# Racial and Ethnic Disparities in the Management of Diabetic Feet

**DOI:** 10.1007/s12178-023-09867-7

**Published:** 2023-09-21

**Authors:** Elizabeth O. Clayton, Confidence Njoku-Austin, Devon M. Scott, Jarrett D. Cain, MaCalus V. Hogan

**Affiliations:** Department of Orthopaedic Surgery, University of Pittsburgh, 3471 Fifth Ave., Suite #911, Pittsburgh, PA 15213 USA

**Keywords:** Diabetes, Diabetic foot ulcer, Race, Disparities, Health equity

## Abstract

**Purpose of Review:**

Diabetes mellitus is a chronic medical condition affecting many individuals worldwide and leads to billions of dollars spent within the healthcare system for its treatment and complications. Complications from diabetes include diabetic foot conditions that can have a devasting impact on quality of life. Diabetic foot ulcers and amputations occur in minority individuals at an increased rate compared to White individuals. This review provides an update examining the racial and ethnic disparities in the management of diabetic foot conditions and the differences in rates of amputation.

**Recent Findings:**

Current research continues to show a disparity as it relates to diabetic foot management. There are novel treatment options for diabetic foot ulcers that are currently being explored. However, there continues to be a lack in racial diversity in new treatment studies conducted in the USA.

**Summary:**

Individuals from racial and ethnic minority groups have diabetes at higher rates compared to White individuals, and are also more likely to develop diabetic foot ulcers and receive amputations. Over the last few years, more efforts have been made to improve health disparities. However, there needs to be an improvement in increasing racial diversity when investigating new therapies for diabetic foot ulcers.

## Introduction

Diabetes mellitus is a chronic illness often accompanied by peripheral vascular disease affecting many individuals worldwide. By 2040, it is projected that 642 million people on the planet will have diabetes, about 10% of the world’s population [1•]. The risk of patients with diabetes developing diabetic foot ulcers (DFU) is 15 to 25%, with reoccurrence rates at 5 years increasing dramatically, up to 50 to 70% [2]. In the USA, billions of dollars are spent in direct costs, for the treatment of diabetes and its complications, and in indirect costs, such as a patient’s inability to work, leading to disability and premature mortality [3]. About 20% of severe diabetic foot infections result in some type of amputation. Patients with diabetes who have had a foot ulcer are two times more likely to die at ten years compared to patients with diabetes who have never had an ulcer [4]. There is an increased prevalence of diabetes among individuals from minority groups, and research continues to demonstrate differences in rates of DFU and amputation compared to White individuals [5••].

## Race, Ethnicity, and Disparity

In discussing racial and ethnic disparities in managing diabetic feet, the concepts of race and health disparities must be explored. Race is a social construct and does not have a scientific foundation [6]. It groups individuals based on physical characteristics like skin color through self-identification and by third-party observation [7]. Moreover, ethnicity encompasses the cultural norms, values, and behaviors that connect groups based on shared culture and language [8•].

Based on the definition provided by the Centers for Disease Control and Prevention, health disparities “are differences in health outcomes between groups that reflect social inequalities” [9]. While there is no genetic basis for defining race, race and ethnicity are essential to consider because they significantly influence racial stratification, which plays a role in access to care, treatment, bias, and racial discrimination and racism [8•]. These factors contribute to the health disparities that exist and, thus, the differences in the management of diabetic feet among different racial and ethnic groups.

## Racial Disparities in Diabetic Feet Prevalence, Incidence, Risk Factors, and Mortality

Racial and ethnic minorities have a higher rate of diabetes than White people [5••]. White adults have the lowest prevalence of diabetes; the prevalence is estimated at 7.3% for females and 9.4% for males. For African American adults, the estimated prevalence is 14.7% for females and 13.4% for males. Hispanic adults have a prevalence of 15.1% and 14.1%, and for Asian adults, the rates are 12.8% and 9.9% for males and females, respectively. Native American and Alaska Native adults have the highest prevalence, 14.9% and 15.3% for males and females, respectively [10•]. Therefore, there is a higher incidence of DFUs and amputations in individuals from minority backgrounds [11].

Diabetic foot osteomyelitis occurs in about 20 to 60% of DFUs [12•]. According to Winkler et al., who conducted a retrospective study with 583 patients who developed diabetic foot osteomyelitis, they found that hindfoot diabetic osteomyelitis increases the risk for a major amputation five fold when compared to forefoot diabetic osteomyelitis [12•].

According to a review completed by Rossboth et al., the risk factors that have been associated with developing diabetic foot diseases across the literature include male gender, poor glycemic control, peripheral neuropathy, retinopathy and nephropathy, insulin use, duration of diabetes, smoking, and height. The authors concluded that glycemic control and smoking are the modifiable risk factors associated with diabetic feet continuously cited in studies [13•].

## Racial Disparities in Diabetic Feet Management

Many studies have stressed the importance of using a multidisciplinary team in leading to better outcomes for patients with diabetic foot conditions [5••, 14•, 15]. Diabetic foot conditions include foot ulcerations, Charcot neuroarthropathy, infection, and osteomyelitis, which require antimicrobial treatment, debridement, the need for hospitalization, revascularization procedures, and, unfortunately, in extreme cases, amputation (Table 1). Other techniques used to improve oxygenation and promote healing in diabetic foot conditions include hyperbaric oxygen therapy, far infrared energy, recombinant proteins and growth factors, and using biomaterials like self-assembling peptides [16, 17•, 18, 19•]. Methods have also been investigated to promote the healing of chronic wounds using different types of dressings such as hydrogel dressings, film dressings, foam dressings, hydrocolloid dressings, and alginate dressings that have various levels of efficacy [20••].

**Table 1:** Diabetic foot conditions and management

Diabetic foot conditions	Pathophysiology	Management
Ulceration	Multifactorial process due to complications of neuropathy (peripheral, autonomic, and motor), vascular disease, immunodeficiency, and uncontrolled blood glucose	Local debridement, nonweightbearing status, and frequent dressing
Charcot neuroarthropathy	Unclear, theories include neurovascular dysregulation (an increase in blood flow leads to damage to the somatic nerves), repeated trauma to joints leading to fractures and deformation, and the relationship between diabetes, osteoporosis, and regulatory factors	Anti-osteoporotic drugs and amputation if complicated by development of an ulcer
Infection	Due to risk factors including deep ulcers, ulcers existing for > 30 days, history of recurrent ulcers, traumatic injuries, and concurrent vascular disease	Hospitalization with consistent monitoring of lab values (e.g., CBC, ESR, CRP, renal, and hepatic function) *Superficial:* surgical debridement, moist dressing, and nonweightbearing status + antibiotics until resolution *Moderate:* immediate hospitalization *Severe:* mandatory early surgical intervention to drain abscesses and removal of necrotic tissue
Osteomyelitis	Tissue infection that progresses to involvement of periosteum, cortex, and medullary bone	Hospitalization and extensive monitoring with resection of infected bones or proximal-level amputation + antibiotic therapy

Diabetic foot ulceration is a multifactorial process due to complications of neuropathy (peripheral, autonomic, and motor), vascular disease, immunodeficiency, and uncontrolled blood glucose and occurs in 15% of patients with diabetes. DFUs is often managed with local debridement, nonweightbearing status, and frequent dressing [1•].

Unfortunately, patients with diabetes have hypoimmunity, and diabetic foot ulcers often lead to infection [20••]. Diabetic foot infections are due to risk factors such as deep ulcers, ulcers existing for more than 30 days, a history of recurrent ulcers, traumatic injuries, and concurrent vascular disease [1•]. Often resulting in hospitalization, these patients must undergo consistent monitoring of lab values (e.g., CBC, ESR, CRP, renal and hepatic function). Based on the severity of the infection, the steps taken can vary. Superficial infections involve surgical debridement, moist dressing, and nonweightbearing status with antibiotics until resolution. Treatment for moderate infections requires an escalation in management, prompting immediate hospitalization plus the protocols for superficial infections. Severe infections include all the treatment modalities, with early surgical intervention being pivotal [1•].

Osteomyelitis develops when a diabetic foot infection has progressed to involve the periosteum, cortex, and medullary bone. This requires hospitalization and extensive monitoring with resection of infected bones or proximal-level amputation with antibiotic therapy [21•].

A lack of access to care for racial and ethnic minorities influences the increase in prevalence of ulcer diagnosis at a more severe stage and the risk of hospitalization for diabetic foot ulcer. When controlling for DFU incidence, Black and Hispanic individuals are less likely to receive revascularization procedures, benefit from limb preservation efforts, and more likely to receive amputations than White individuals [9].

Diabetic foot infection is a serious complication and accounts for half of all cases of lower limb amputations [22]. Therefore, ongoing research is being completed to advance the treatments for diabetic foot conditions. Mahdipour et al. attempted to analyze the roles of recombinant proteins and growth factors in managing diabetic foot ulcers to evaluate their efficacy. The authors concluded that the epidermal growth factor showed the most use in improving the healing of diabetic foot ulcers but also asserted that the studies examined contained methodological flaws, making it difficult to ascertain clear conclusions [23•].

Given the devasting effects diabetic foot conditions have on both the quality of life of patients and the healthcare system, there is a crucial push to develop and explore innovative treatments to improve the outcomes of these morbid conditions. Despite the emphasis in recent years to address racial and ethnic disparities, there continues to be a gap in research studies to create more racially diverse research cohorts, and new treatments for diabetic foot conditions are no exception [24]. A study conducted by Athonvaragnkul et al. investigated the role of far infrared energy in improving peripheral circulation with a cohort of 32 participants, with only five being non-White [17•]. While this is one of the few studies conducted in the USA that explore new therapies, there are distinct social and economic factors to consider based on the history of this country [25••]. Therefore, it is vital to acknowledge the groups that are disproportionately affected by more severe presentations of diabetic foot conditions and make efforts to include those individuals in novel studies. It is imperative to emphasize that there are no clear guidelines on using race, ethnicity, and ancestry [26••]. Including these groups is not to compare the biological or genetic differences related to new treatment but rather to be more mindful of the impact that race and ethnicity have on access to resources and treatment.

## Racial Disparities in Amputation Rates

A lower extremity is amputated due to diabetes every 20 s [27•]. Amputations of the lower limb negatively affect a patient’s quality of life and are a tremendous financial burden, including costs for rehabilitation, prosthetic creation, management, and maintenance. While this can be difficult for any patient, it is more devasting for individuals from vulnerable populations as it significantly changes the psychosocial, functional, and economic aspects of their lives [28]. Lower extremity amputations (LEA) occur at higher rates in racial and ethnic minority groups, for people with low socioeconomic status, and in geographically vulnerable areas like rural areas without access to specialist care [5••].

As it relates to amputations secondary to diabetic complications, it is well documented that health disparities exist among individuals in racial and ethnic minority groups. Diabetic-related amputations are two to four times more likely to occur in Black, Native American, Hispanic, and other ethnic minority groups than in non-Hispanic White patients [5••]. They are more likely to have complications and a lower survival rate [25••]. Studies have been conducted to analyze the differences in rates and levels of amputation (Table 2).

**Table 2:** Amputation rates among different minority and ethnic groups

Study	Author(s)	Population	Level of amputation	Increased rate of amputation among minority race and ethnicity
Variation in the incidence and proportion of diabetes-related amputations in minorities	Lavery et al. (1996) [29]	8169 hospitalizations for LEA in African Americans, Hispanics, and non-Hispanic White	More proximal amputations were seen in African Americans compared to Hispanic and non-Hispanic White people (*p* < 0.001)	More amputations in African American people (61.6%) and Hispanic people (82.7%) than in non-Hispanic White people (56.8%) (*p* < 0.001)
Diabetes mellitus and nontraumatic lower extremity amputation in black and White Americans: the National Health and Nutrition Examination Survey Epidemiologic Follow-up Study, 1971–1992	Resnick et al. (1999) [32]	14,407 subjects in the National Health and Nutrition Examination Survey Epidemiologic Follow-up Study	Locations of LEAs were distributed similarly between White and Black participants	Black participants with diabetes were more likely to receive LEAs than White participants but the difference is only significant for incidence of diabetes mellitus and not prevalence of diabetes mellitus (3.4% vs. 1.4%, *p* = 0.02)
Effects of ethnicity and nephropathy on lower-extremity amputation risk among diabetic veterans	Young et al. (2003) [33]	3289 individuals had an LEA within the U.S. Veterans Affairs (VA) Health Care System during 1998	Asian participants were more likely to receive toe amputations than White and other participants; Native American participants were more likely to receive BKAs	Native American participants (RR 1.74, 95% CI 1.39–2.18), followed by Black (RR 1.41, 95% CI 1.34–1.48) and Hispanic participants (RR 1.28, 95% CI 1.20–1.38), had the highest risk of LEA compared with White participants
Racial differences in primary and repeat amputation: a multihospital study	Feinglass et al. (2005) [34]	513 patients with 71% undergoing a major amputation	African American people without diabetes have greater racial and ethnic differences in amputation level than those among patients with diabetes	65.9% of African American patients with diabetes (OR 1.69, 95% CI 1.11–2.56) compared to 53.1% White or other-race patients underwent primary amputations
Association between race/ethnicity and the risk of amputation of lower extremities among Medicare beneficiaries with diabetic foot ulcers and diabetic foot infections	Tan et al. (2020) [35•]	92,929 Medicare beneficiaries diagnosed with DFUs and/or DFIs	Not mentioned	Black (HR 1.9, 95% CI 1.7–2.2) and Native American (HR 1.8, 95% CI 1.3–2.6) beneficiaries were at an increased risk of major amputation compared to White beneficiaries
Racial, rural, and regional disparities in diabetes-related lower-extremity amputation rates, 2009–2017	Akinlotan et al. (2021) [36•]	112,713 patients with diabetes-related discharges in the US had a minor or major LEA	Native Americans were more likely to have a minor (toe and foot) amputation compared with White (OR 1.26; 95% CI 1.167–1.36)	Black (OR 1.12, 95% CI 1.08–1.16), Hispanic (OR 1.24, 95% CI 1.19–1.29), and Native American (OR 1.32, 95% CI 1.16–1.50) patients were more likely to experience a major amputation compared with White
Racial disparities in health care with timing to amputation following diabetic foot ulcer	Miller et al. (2022) [25••]	68,633 Medicare fee-for-service beneficiaries with a DFU who experienced a LEA within the 5-year study time	Not mentioned	There was an increase in the proportion of Black/African American individuals who received an LEA after DFU; they are more likely to have an amputation within the first year after a DFU compared to non-Hispanic White individuals (OR 2.18, 95% CI 2.13–2.23)

*LEA*, lower extremity amputation; *OR*, odds ratio; *CI*, confidence interval; *DFU*, diabetic foot ulcer; *DFI*, diabetic foot infection; *HR*, hazard ratio

Lavery et al. studied 8169 hospitalizations for LEA in African Americans, Hispanics, and non-Hispanic White, finding that more amputations occurred in African American people (61.6%) and Hispanic people (82.7%) than in non-Hispanic White people (56.8%) (*p* < 0.001). The authors also stated that more proximal amputations were seen in African Americans compared to Hispanic and non-Hispanic White people (*p* < 0.001) [29]. It is important to note that more proximal amputations require a higher demand on the cardiovascular and pulmonary systems for prosthetic gait [30•]. There is also a strong correlation between the level of amputation and prosthesis use. Individuals with below-knee amputations are more likely to ambulate with or without a prosthesis than those with above-knee amputations [31•]. The capacity to regain the skill of walking is vital to improving a person’s quality of life, such as their ability to participate in social activities and prevent metabolic bone disease due to immobility [30•].

The disparities among African American individuals compared to White individuals were consistent with the results concluded by Resnick et al., analyzing 14,407 subjects in the National Health and Nutrition Examination Survey Epidemiologic Follow-up Study. Black participants with diabetes were more likely to receive LEAs than White participants but the difference is only significant for the incidence of diabetes mellitus (3.4% vs. 1.4%, *p* = 0.02) as opposed to the prevalence of diabetes mellitus, which was not statistically significant. The study also showed that the locations of LEAs were distributed similarly between White and Black participants [32].

Young et al. documented 3289 individuals had an LEA within the U.S. Veterans Affairs (VA) Health Care System during 1998. Of the patients included in the study, Native American participants (RR 1.74, 95% CI 1.39–2.18), followed by Black (RR 1.41, 95% CI 1.34–1.48), and Hispanic participants (RR 1.28, 95% CI 1.20–1.38), had the highest risk of LEA compared with White participants. Regarding the level of amputation, it was noted that Asian participants were more likely to receive toe amputations than White and other participants; Native American participants were more likely to receive BKAs [33].

The results of a study completed by Feinglass et al. showed that 65.9% of African American patients with diabetes (OR 1.69, 95% CI 1.11–2.56) compared to 53.1% White or other race patients underwent primary amputations. The authors concluded that because African American people without diabetes have greater racial and ethnic differences in amputation levels than those among patients with diabetes, it demonstrates that both diabetic and non-diabetic African American individuals who undergo amputation are at equally increased risk for primary and repeat amputation and supports the conclusion that diabetes prevalence does not drive racial differences [34].

Tan et al. reviewed 92,929 Medicare beneficiaries with DFUs and/ or DFIs. Black (HR 1.9, 95% CI 1.7–2.2) and Native American (HR 1.8, 95% CI 1.3–2.6) beneficiaries were at an increased risk of major amputation compared to White beneficiaries. The authors discovered that beneficiaries diagnosed by a podiatrist or primary care physician or at an outpatient visit were less likely to receive a major LEA [35•].

From 2009 to 2017, there were 112,713 patients with diabetes-related discharges in the USA had a minor or major LEA. Black (OR 1.12, 95% CI 1.08–1.16), Hispanic (OR 1.24, 95% CI 1.19–1.29), and Native American (OR 1.32, 95% CI 1.16–1.50) patients were more likely to experience a major amputation compared with White. Native Americans were more likely to have a minor (toe and foot) amputation compared with White (OR 1.26; 95% CI 1.167–1.36) [36•].

Miller et al. evaluated 68,633 Medicare fee-for-service beneficiaries with a DFU who experienced an LEA within a 5-year study time and discovered that there was an increase in the proportion of Black/African American individuals who received an LEA after DFU; they are more likely to have an amputation within the first year after a DFU compared to non-Hispanic White individuals (OR 2.18, 95% CI 2.13–2.23) [25••].

In summary, there continues to be differences in amputation rates between racial and ethnic minority groups. Non-White individuals are more likely to receive more proximal or aggressive amputations than White individuals, regardless of diabetes mellitus status. African American patients without diabetes mellitus continue to be at an increased risk of primary and subsequent amputations compared to White individuals. These findings highlight the increased amputation rates among African American and other non-White racial groups compared to White individuals, even after excluding diabetes prevalence.

## Future Directions

As with any medical condition, it is important that diabetic foot conditions are managed using a multidisciplinary approach [20••]. Recently, qualitative studies have been conducted to develop a deeper understanding as to why the disparities in diabetic foot conditions exist, and what the barriers are to obtaining proper care among minority groups. This is a multi-level issue that requires intervention at various points of entry within the healthcare system. Efforts should be made to improve health literacy, relationships with providers, and access to quality and effective medical care and services [37••].

## Conclusions

While there continues to be improvement in the management of diabetic foot, there is a dire need to address the racial and ethnic disparities that exist. Future efforts must be made to improve the diversity in patient participation when investigating novel approaches to treat diabetic foot ulcers and other conditions.

## References

[1] • Ferreira RC. Diabetic foot. Part 1: ulcers and Infections. Rev Bras Ortop (Sao Paulo). 2020;55(4):389–396. 10.1055/s-0039-3402462. **Review that outlines the pathophysiology and treatment of diabetes complications affecting the feet, such as ulcers and secondary infections, highlighting the importance of clinical history and physical examination in accurate diagnosis.** 10.1055/s-0039-3402462 PMC7494373 32968329

[2] Hines A, Alavi A, Davis MDP (2021). Cutaneous manifestations of diabetes. Med Clin North Am.

[3] Driver VR, Fabbi M, Lavery LA, Gibbons G (2010). The costs of diabetic foot: the economic case for the limb salvage team. J Vasc Surg.

[4] Armstrong DG, Boulton AJM, Bus SA (2017). Diabetic foot ulcers and their recurrence. N Engl J Med.

[5] McDermott K, Fang M, Boulton AJM, Selvin E, Hicks CW (2023). Etiology, epidemiology, and disparities in the burden of diabetic foot ulcers. Diabetes Care.

[6] Witzig R (1996). The medicalization of race: scientific legitimization of a flawed social construct. Ann Intern Med.

[7] Borrell LN (2005). Racial identity among Hispanics: implications for health and well-being. Am J Public Health.

[8] Borrell LN, Elhawary JR, Fuentes-Afflick E, Witonsky J, Bhakta N, Wu AHB, Bibbins-Domingo K, Rodríguez-Santana JR, Lenoir MA, Gavin JR, Kittles RA, Zaitlen NA, Wilkes DS, Powe NR, Ziv E, Burchard EG (2021). Race and genetic ancestry in medicine - a time for reckoning with racism. N Engl J Med.

[9] Frieden TR; Centers for Disease Control and Prevention (CDC). Forward: CDC health disparities and inequalities report - United States, 2011. MMWR Suppl. 2011 14;60(1):1–2 21430612

[10] • Barnes JA, Eid MA, Creager MA, Goodney PP. Epidemiology and risk of amputation in patients with diabetes mellitus and peripheral artery disease. Arterioscler Thromb Vasc Biol. 2020;40(8):1808–1817. 10.1161/ATVBAHA.120.314595. **Review detailing the epidemiology of diabetes and peripheral vascular disease and the risk of amputation in the United States and its distribution based on regional, racial/ethnic, and socioeconomic characteristics.** 10.1161/ATVBAHA.120.314595 PMC7377955 32580632

[11] Margolis DJ, Malay DS, Hoffstad OJ, Leonard CE, MaCurdy T, López de Nava K, Tan Y, Molina T, Siegel KL. Prevalence of diabetes, diabetic foot ulcer, and lower extremity amputation among Medicare beneficiaries, 2006 to 2008: Data Points #1. 2011 Feb 17. In: Data Points Publication Series [Internet]. Rockville (MD): Agency for Healthcare Research and Quality (US); 2011 22049561

[12] Winkler E, Schöni M, Krähenbühl N, Uçkay I, Waibel FWA (2022). Foot osteomyelitis location and rates of primary or secondary major amputations in patients with diabetes. Foot Ankle Int.

[13] • Rossboth S, Lechleitner M, Oberaigner W. Risk factors for diabetic foot complications in type 2 diabetes-a systematic review. Endocrinol Diabetes Metab. 2020 17;4(1):e00175. 10.1002/edm2.175. **Review assessing risk factors associated with diabetic foot complications, finding that glycemic control and smoking are consistent positive associations with diabetic foot syndrome.** 10.1002/edm2.175 PMC7831214 33532615

[14] Gazzaruso C, Gallotti P, Pujia A, Montalcini T, Giustina A, Coppola A (2021). Predictors of healing, ulcer recurrence and persistence, amputation and mortality in type 2 diabetic patients with diabetic foot: a 10-year retrospective cohort study. Endocrine.

[15] Chen HF, Ho CA, Li CY (2006). Age and sex may significantly interact with diabetes on the risks of lower-extremity amputation and peripheral revascularization procedures: evidence from a cohort of a half-million diabetic patients. Diabetes Care.

[16] Capó X, Monserrat-Mesquida M, Quetglas-Llabrés M, Batle JM, Tur JA, Pons A, Sureda A, Tejada S (2023). Hyperbaric oxygen therapy reduces oxidative stress and inflammation, and increases growth factors favouring the healing process of diabetic wounds. Int J Mol Sci.

[17] • Athonvarangkul D, Wang K, Deng Y, Inzucchi SE, Mayerson A. Improved extremity tissue oxygenation with short-term exposure to textiles embedded with far infrared light emitting thermoactive particles in patients with diabetes mellitus. Diab Vasc Dis Res. 2023;20(2):14791641231170282. 10.1177/14791641231170282. **Prospective randomized control trial using far infrared to examine its efficacy in improving peripheral circulation which revealed effectiveness in the short-term.** 10.1177/14791641231170282 PMC10123901 37073436

[18] Pérez-Panero AJ, Ruiz-Muñoz M, Cuesta-Vargas AI, Gónzalez-Sánchez M. Prevention, assessment, diagnosis and management of diabetic foot based on clinical practice guidelines: a systematic review. Medicine (Baltimore). 2019;98(35):e16877. 10.1097/MD.0000000000016877 10.1097/MD.0000000000016877 PMC6736276 31464916

[19] • Guan T, Li J, Chen C, Liu Y. Self-assembling peptide-based hydrogels for wound tissue repair. AdvSci (Weinh). 2022;9(10):e2104165. 10.1002/advs.202104165. **Review outlining the advantages of self-assembling peptide-based hydrogels to promote wound healing and recommending the importance for future studies looking at its practical applications.** 10.1002/advs.202104165 PMC8981472 35142093

[20] Yang L, Rong GC, Wu QN (2022). Diabetic foot ulcer: challenges and future. World J Diabetes.

[21] Sohrabi K, Belczyk R (2022). Surgical treatment of diabetic foot and ankle osteomyelitis. Clin Podiatr Med Surg.

[22] Tchero H, Kangambega P, Fluieraru S, Bekara F, Teot L. Management of infected diabetic wound: a scoping review of guidelines. F1000Res. 2019;8:737. 10.12688/f1000research.18978.1 10.12688/f1000research.18978.1 PMC7265589 32528649

[23] Mahdipour E, Sahebkar A (2020). The role of recombinant proteins and growth factors in the management of diabetic foot ulcers: a systematic review of randomized controlled trials. J Diabetes Res.

[24] Konkel L (2015). Racial and ethnic disparities in research studies: the challenge of creating more diverse cohorts. Environ Health Perspect.

[25] •• Miller TA, Campbell JH, Bloom N, Wurdeman SR. Racial disparities in health care with timing to amputation following diabetic foot ulcer. Diabetes Care. 2022 1;45(10):2336–2341. 10.2337/dc21-2693. **Retrospective cohort study of enrolled Medicare fee-for-service beneficiaries with diabetic foot ulcers demonstrated that Black beneficiaries had almost two times the odds of receiving a lower-limb amputation than White beneficiaries.** 10.2337/dc21-2693 PMC9862414 36069831

[26] Mauro M, Allen DS, Dauda B, Molina SJ, Neale BM, Lewis ACF (2022). A scoping review of guidelines for the use of race, ethnicity, and ancestry reveals widespread consensus but also points of ongoing disagreement. Am J Hum Genet.

[27] Edmonds M, Manu C, Vas P (2021). The current burden of diabetic foot disease. J Clin Orthop Trauma.

[28] Lefebvre KM, Lavery LA (2011). Disparities in amputations in minorities. Clin Orthop Relat Res.

[29] Lavery LA, Ashry HR, van Houtum W, Pugh JA, Harkless LB, Basu S (1996). Variation in the incidence and proportion of diabetes-related amputations in minorities. Diabetes Care.

[30] • Damiani C, Pournajaf S, Goffredo M, Proietti S, Denza G, Rosa B, Franceschini M, Casale R. Community ambulation in people with lower limb amputation: an observational cohort study. Medicine (Baltimore). 2021;100(3):e24364. 10.1097/MD.0000000000024364. **Observational cohort study investigating factors associated with community ambulation based on socio-demographic and clinical factors.** 10.1097/MD.0000000000024364 PMC7837990 33546072

[31] • Pran L, Harnanan D, Baijoo S, Short A, Cave C, Maharaj R, Cawich SO, Naraynsingh V. Major lower limb amputations: recognizing pitfalls. Cureus. 2021;13(8):e16972. 10.7759/cureus.16972. **Retrospective study highlighting the low prosthetic limb acquisition for a patient population in Trinidad and Tobago.** 10.7759/cureus.16972 PMC8423325 34540383

[32] Resnick HE, Valsania P, Phillips CL (1999). Diabetes mellitus and nontraumatic lower extremity amputation in black and white Americans: the National Health and Nutrition Examination Survey Epidemiologic Follow-up Study, 1971–1992. Arch Intern Med.

[33] Young BA, Maynard C, Reiber G, Boyko EJ (2003). Effects of ethnicity and nephropathy on lower-extremity amputation risk among diabetic veterans. Diabetes Care.

[34] Feinglass J, Rucker-Whitaker C, Lindquist L, McCarthy WJ, Pearce WH (2005). Racial differences in primary and repeat lower extremity amputation: results from a multihospital study. J Vasc Surg.

[35] • Tan TW, Armstrong DG, Concha-Moore KC, Marrero DG, Zhou W, Calhoun E, Chang CY, Lo-Ciganic WH. Association between race/ethnicity and the risk of amputation of lower extremities among medicare beneficiaries with diabetic foot ulcers and diabetic foot infections. BMJ Open Diabetes Res Care. 2020;8(1):e001328. 10.1136/bmjdrc-2020-001328. **Retrospective cohort study shows that racial and ethnic disparities in rates of amputations exist in populations of fee-for-service Medicare beneficiaries.** 10.1136/bmjdrc-2020-001328 PMC7449291 32843499

[36] Akinlotan MA, Primm K, Bolin JN, Ferdinand Cheres AL, Lee J, Callaghan T, Ferdinand AO (2021). Racial, rural, and regional disparities in diabetes-related lower-extremity amputation rates, 2009–2017. Diabetes Care.

[37] Tan TW, Crocker RM, Palmer KNB, Gomez C, Armstrong DG, Marrero DG (2022). A qualitative study of barriers to care-seeking for diabetic foot ulceration across multiple levels of the healthcare system. J Foot Ankle Res.

