# Correction to: Racial and Ethnic Disparities in the Management of Diabetic Feet

**DOI:** 10.1007/s12178-024-09882-2

**Published:** 2024-01-25

**Authors:** Elizabeth O. Clayton, Confidence Njoku-Austin, Devon M. Scott, Jarrett D. Cain, MaCalus V. Hogan

**Affiliations:** Department of Orthopaedic Surgery, University of Pittsburgh, 3471 Fifth Ave., Suite #911, Pittsburgh, PA 15213 USA

**Correction to: Current Reviews in Musculoskeletal Medicine (2023) 16:550-556**

10.1007/s12178-023-09867-7

In this article the term “Caucasian” should have been “White.”

The original article has been corrected.

